# Phosphate transporters in marine phytoplankton and their viruses: cross-domain commonalities in viral-host gene exchanges

**DOI:** 10.1111/j.1462-2920.2011.02576.x

**Published:** 2012-01

**Authors:** Adam Monier, Rory M Welsh, Chelle Gentemann, George Weinstock, Erica Sodergren, E Virginia Armbrust, Jonathan A Eisen, Alexandra Z Worden

**Affiliations:** 1 Monterey Bay Aquarium Research Institute7700 Sandholdt Road, Moss Landing, CA 95039, USA; 2 Remote Sensing Systems444 Tenth Street, Suite 200, Santa Rosa, CA, 95401, USA; 3 The Genome Center, Washington University School of Medicine4444 Forest Park Avenue, St. Louis, MO 63108, USA; 4 School of Oceanography, University of WashingtonSeattle, WA 98195, USA; 5 University of California DavisDavis, CA 95616DOE Joint Genome Institute Walnut CreekCA, USA

## Abstract

Phosphate (PO_4_) is an important limiting nutrient in marine environments. Marine cyanobacteria scavenge PO_4_ using the high-affinity periplasmic phosphate binding protein PstS. The *pstS* gene has recently been identified in genomes of cyanobacterial viruses as well. Here, we analyse genes encoding transporters in genomes from viruses that infect eukaryotic phytoplankton. We identified inorganic PO_4_ transporter-encoding genes from the PHO4 superfamily in several virus genomes, along with other transporter-encoding genes. Homologues of the viral *pho4* genes were also identified in genome sequences from the genera that these viruses infect. Genome sequences were available from host genera of all the phytoplankton viruses analysed except the host genus *Bathycoccus*. *Pho4* was recovered from *Bathycoccus* by sequencing a targeted metagenome from an uncultured Atlantic Ocean population. Phylogenetic reconstruction showed that *pho4* genes from pelagophytes, haptophytes and infecting viruses were more closely related to homologues in prasinophytes than to those in what, at the species level, are considered to be closer relatives (e.g. diatoms). We also identified PHO4 superfamily members in ocean metagenomes, including new metagenomes from the Pacific Ocean. The environmental sequences grouped with pelagophytes, haptophytes, prasinophytes and viruses as well as bacteria. The analyses suggest that multiple independent *pho4* gene transfer events have occurred between marine viruses and both eukaryotic and bacterial hosts. Additionally, *pho4* genes were identified in available genomes from viruses that infect marine eukaryotes but not those that infect terrestrial hosts. Commonalities in marine host-virus gene exchanges indicate that manipulation of host-PO_4_ uptake is an important adaptation for viral proliferation in marine systems. Our findings suggest that PO_4_-availability may not serve as a simple bottom-up control of marine phytoplankton.

## Introduction

Marine phytoplankton play a major role in global photosynthesis and CO_2_ capture from Earth's atmosphere (Field *et al*., 1998). Phytoplankton populations are controlled by grazing and viral mortality as well as nutrient availability and other biological and physico-chemical factors. How these different forces control individual taxa remains poorly understood, see, e.g. (Azam *et al*., 1983; Rohwer and Thurber, 2009). A major challenge for understanding eukaryotic phytoplankton controls is their tremendous diversity and the fact that many remain uncultured. The smallest of these are the picoeukaryotes (≤ 2–3 µm diameter), a broad assemblage composed of picoprasinophytes, small haptophytes and stramenopiles (Worden and Not, 2008; Shi *et al*., 2009; Cuvelier *et al*., 2010). Picoprasinophytes in the order Mamiellales are widespread and genome sequences are available for two common genera, *Ostreococcus* (Derelle *et al*., 2006; Palenik *et al*., 2007) and *Micromonas* (Worden *et al*., 2009), but not yet for *Bathycoccus*. *Ostreococcus* is seen in tropical and temperate waters while *Bathycoccus* and *Micromonas* extend to polar systems (Not *et al*., 2005; Worden *et al*., 2009). While complete genome sequences are not yet available for haptophyte and stramenopilepicoplankton, the picoprasinophyte genomes provide information on the molecular underpinnings of nutrient uptake and insights on the relative importance of bottom-up controls.

Concentrations of the major nutrients nitrate, ammonium and phosphate (PO_4_) vary seasonally in the euphotic zone where they influence the dynamics and successional patterns of resident phytoplankton communities. PO_4_ depletion in particular is thought to cause intense competition between different taxa in the North Atlantic Ocean and to have influenced gene content in different strains of the picocyanobacteria *Prochlorococcus* and *Synechococcus* through selective adaptations (Scanlan *et al*., 2009). Indeed, *Prochlorococcus* populations were recently reported to have different frequencies of PO_4_-uptake related genes depending on whether they were from the North Pacific or the North Atlantic Gyre characterized by much lower PO_4_ concentrations (Coleman and Chisholm, 2010). Similar results were reported for the SAR11 clade, a lineage of widely distributed marine heterotrophic bacteria. These findings were hypothesized to reflect the influence of water mass characteristics on the gene repertoires of resident taxa (Coleman and Chisholm, 2010).

The two main types of inorganic PO_4_ transporters in cultured microbial taxa are encoded by genes in the PHO4 superfamily (Pfam PF01384) and Pst genes. Many eukaryotes and bacteria encode PHO4 superfamily members. This superfamily includes high- and low-affinity inorganic phosphate transporters. In eukaryotic phytoplankton a *pho4* gene has been identified in expressed sequence tags from the haptophyte *Emiliania huxleyi* (Dyhrman *et al*., 2006a) as well as in the genome sequence of a virus that infects it, EhV-86 (Wilson *et al*., 2005). Comparative analysis of putative transporter-encoding genes in *Micromonas* and *Ostreococcus* indicate that PHO4 superfamily members are present in these taxa as well (Worden *et al*., 2009), but their relatedness and PO_4_ affinities are not yet known. The marine picocyanobacteria use the Pst system, a multi-unit high-affinity inorganic PO_4_ transport system that depends on the high-affinity periplasmic PO_4_-binding protein encoded by the gene *pstS*/*phoS* (Diaz *et al*., 2005). *PstS* homologues are present in all sequenced *Prochlorococcus* and *Synechococcus* genomes, sometimes in multiple copies (Martiny *et al*., 2006; Scanlan *et al*., 2009). Interestingly, some marine cyanophages also encode *pstS* genes and the presence of *pstS* in cyanophage genomes appears to be linked to whether the phage source waters were PO_4_-deplete or not (Sullivan *et al*., 2005; Sullivan *et al*., 2010). Phage-mediated gene transfer has been hypothesized to be responsible for the fact that many PO_4_-uptake related genes in *Prochlorococcus* ecotypes do not appear to follow the ‘species’ tree (Martiny *et al*., 2006).

Here, we analyse phosphate and other transporter-encoding genes in published genomes from viruses that infect eukaryotic phytoplankton. Ecological and evolutionary aspects of PHO4 superfamily members were investigated in the viruses and their hosts, including a ‘wild’*Bathycoccus* population. The distribution and diversity of this transporter gene was evaluated in metagenomes from a North Pacific Ocean transect and other marine environments.

## Results and discussion

### Virally encoded transporter genes

We analysed viruses infecting unicellular and/or marine eukaryotes for transporter-encoding genes, including the *pho4* gene previously reported in EhV-86 (Wilson *et al*., 2005). Analysis of open reading frames (ORFs) in published viral genomes showed that several eukaryotic viruses encoded *pho4* genes, as well as other putative transporter genes (Table 1). Apart from the amoeba-infecting mimivirus, all of these viruses belong to the Phycodnaviridae (Wilson *et al*., 2005; Derelle *et al*., 2008; Weynberg *et al*., 2009; 2011; Moreau *et al*., 2010), a family of nucleocytoplasmic large double-stranded DNA viruses. Genes from the different viruses that encoded PHO4 superfamily members had higher overall similarities to each other (ranging from 59% to 94% at the amino-acid level) than did the genes that encoded each of the other transporter types identified in the viral genomes (Table 1). Apart from EhV-86, the other identified *pho4* genes were in Mamiellales-infecting viruses. Specifically, they were found in one of the two viruses infecting *Bathycoccus*, two of the four infecting *Ostreococcus* but not in MpV-1, which infects *Micromonas*. Some of these Mamiellales-infecting viruses contained the *pho4* gene but no other known transporter genes (Table 1). Although genomic data are limited, it is possible that the presence or absence of *pho4* genes reflects the influence of the environment, or environmental stresses acting on their hosts, in shaping viral genomes. For example, the *pho4* gene-encoding virus OtV-2 infects *Ostreococcus* RCC393 (Weynberg *et al*., 2011), a strain that belongs to an *Ostreococcus* clade (OII) known to inhabit warm, oligotrophic waters where PO_4_ is often depleted (Demir-Hilton *et al*., 2011). In contrast, the *Ostreococcus* viruses OtV-1 and OtV-5 do not encode *pho4* and their host, *O. tauri*, appears to be restricted to higher nutrient systems, such as bays, lagoons and brackish waters. Two different *Bathycoccus* viruses, BpV-1 and BpV-2, were isolated against the same *Bathycoccus* strain (RCC1105), but the source waters the viruses came from were collected at different times of the year (fall and winter). Hence, different nutrient conditions associated with seasonal changes may explain the fact that only BpV-1 encodes the *pho4* gene. Different *Micromonas* clades also vary along environmental gradients (Foulon *et al*., 2008) and the distribution of the strain infected by MpV-1 is unknown. Lack of *pho4* in MpV-1 is however consistent with *Micromonas* being most abundant in coastal settings, at least in temperate and subtropical systems (Foulon *et al*., 2008), that are usually PO_4_ replete.

**Table 1:** Transporter and *phoH* sequences detected in all published Mamiellales viruses, other representative eukaryotic viruses and mimivirus (as an outgroup)

		Transporter					Other
			
Virus	Host genus	*PHO4*	*ABC*	*MC*	*MFS*	*VIC*	*phoH*
*Phycodnaviridae*							
BpV-1	*Bathycoccus*	YP_004061633	–	–	–	YP_004061440	YP_004061453
BpV-2	*Bathycoccus*	–	–	–	–	ADQ91178	ADQ91193
MpV-1	*Micromonas*	–	–	–	–	YP_004062056	YP_004062114
OtV-1	*Ostreococcus*	–	–	–	–	–	YP_003494870
OtV-2	*Ostreococcus*	YP_004063655	–	–	–	–	YP_004063457
OtV-5	*Ostreococcus*	–	–	–	–	–	YP_001648107
OlV-1	*Ostreococcus*	YP_004061866	–	–	–	–	YP_004061669
PbCV-1	Chlorella*	–	NP_049022	–	–	NP_048599	–
EhV-86	*Emiliania*	YP_002296186	–	–	YP_293932	–	–
EsV-1	*Ectocarpus*	–	–	–	–	NP_077708	–
FsV	*Feldmannia*	–	–	–	–	–	–
*Mimivirus*							
AcpV-1	Acanthamoeba*	–	YP_003987262	YP_003986777	–	–	–

The asterisk indicates a non-marine host; – indicates not found. Accessions provided were retrieved by blasting predicted ORFs against GenBank NR.

PHO4, inorganic phosphate transporter family (PiT); ABC, ATP-binding cassette superfamily; MC, mitochondrial carrier family; MFS, the major facilitator superfamily; VIC, the voltage-gated ion channel family; phoH, phosphate starvation induced ATPase. BpV-1/2, *Bathycoccus* RCC1105 viruses 1 and 2; MpV-1, *Micromonas pusilla* virus 1; OtV-1/2/5, *Ostreococcus tauri* viruses 1, 2 and 5; OlV-1, *Ostreococcus lucimarinus* virus 1; PbCV-1, *Paramecium bursaria Chlorella* virus 1; EhV-86, *Emiliania huxleyi* virus 86; EsV-1, *Ectocarpus siliculosus* virus 1; FsV, *Feldmannia sp.* virus; mimivirus, *Acanthamoeba polyphaga* mimivirus.

Putative functions of other virally encoded transporters were broader and more difficult to interpret ecologically. A single member of the ATP-Binding Cassette superfamily was found in the *Paramecium bursaria Chlorella* virus (PbCV-1, which infects a *Chlorella* alga that lives in a symbiotic association with *P. bursaria*) and one Major Facilitator Superfamily (small solute transporters) member was identified (Table 1). The latter was found in EhV-86 and an apparently unrelated Major Facilitator Superfamily member has been reported in a dsDNA virus infecting the moth *Helicoverpa zea*. A putative ion channel transporter gene (belonging to the VIC Superfamily) was detected in several of the Mamiellales viruses (Table 1). A homologue of this gene was first reported in PbCV-1 where it was shown to act as a potassium selective channel and appeared to be essential to the viral life cycle (Plugge *et al*., 1999). It was later described in EsV-1, which infects the multicellular brown alga *Ectocarpus siliculosus* (Chen *et al*., 2005). Members of the Cation Channel-forming Heat Shock Protein-70 family were also identified in the *Bathycoccus* viruses (YP_004061438, ADQ91175) and mimivirus (YP_003986897, YP_003986752). A putative ATPase-encoding gene, *phoH*, was also identified in most of the viral genomes (Table 1, see below).

The only viral sequence included in the full PHO4 Pfam alignment at the time of our analysis was from EhV-86. *Pho4* was not present in the 50 nucleocytoplasmic large double-stranded DNA viruses (Asfarviridae, Iridoviridae, Mimiviridae, Phycodnaviridae and Poxviridae) and 46 herpesvirus genomes sequences available when that Pfam alignment was generated. The majority of these viruses infect terrestrial hosts including mammals, non-mammalian vertebrates and invertebrates. Furthermore, *pho4* genes were not detected in newly available genomes from viruses that infect other hosts, including non-marine unicellular eukaryotes. In environments like the mammalian cellular milieu or many freshwater systems, PO_4_ is not considered limiting and therefore selection pressure favouring viruses that encode a PO_4_ transporter (or retention of this gene) is presumably weak. In contrast, manipulation of PO_4_-uptake in marine hosts that often encounter PO_4_-deplete conditions could ensure enough PO_4_ is available for viral replication.

### Origins of virally encoded *pho4* genes

Many previous studies have suggested that viruses acquire genes from their hosts (studies by, for example, Monier *et al*., 2007; Sharon *et al*., 2009; Colson and Raoult, 2010). We screened eukaryotic algal genomes for the *pho4* gene, including hosts for the analysed viruses. In addition to the previously described *E. huxleyi pho4* gene (Dyhrman *et al*., 2006a), genes containing the PHO4 Pfam domain were found in *Aureococcus anophagefferens* (a pelagophyte) and all genome sequenced *Ostreococcus* strains (*O. tauri*, *O. lucimarinus* and *Ostreococcus* RCC809). They were also found in *Micromonas* RCC299 and CCMP1545 but with low similarity to the *Ostreococcus pho4* genes (see discussion below).

**Fig. 1:**
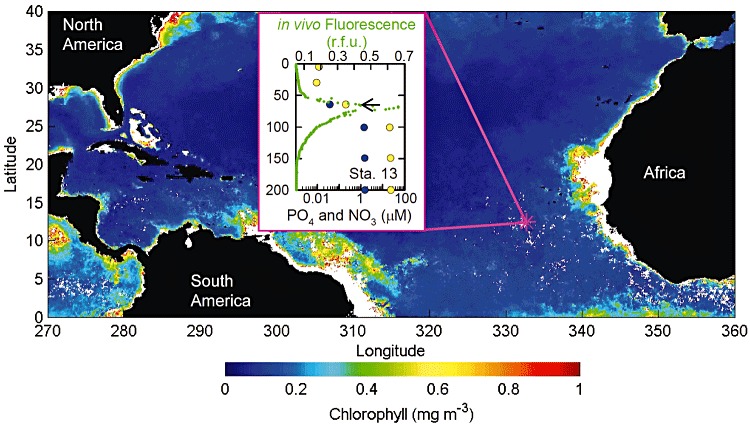
Atlantic Ocean site (pink star) from which a natural population of *Bathycoccus* was sorted and sequenced. Ocean colour represents Aqua MODIS Atlantic Ocean chlorophyll concentration data (mg m^−3^) from 1 May to 5 September 2006, spanning the period of sample collection; black indicates land and white indicates missing data. Lower chlorophyll concentrations reflect lower phytoplankton biomass and occurs in regions with low nutrient availability. (Inset) phosphate (blue) and nitrate plus nitrite (yellow) concentrations as well as *in vivo* fluorescence from chlorophyll (green) at the sort site. The *Y*-axis represents depth (m). Arrow indicates the depth from which *Bathycoccus* was sorted.

To better understand the distribution of *pho4* genes in the Mamiellales and potential gene exchanges between viruses and hosts, we generated and screened a targeted metagenome for *Bathycoccus*. A natural population was sorted by flow cytometry (Fig. S1) from PO_4_-deplete waters (Fig. 1, Table 2) and the 18S rDNA clone library built from the sorted and multiple displacement amplification (MDA)-amplified material was composed solely of *Bathycoccus* sequences (Fig. S2; 99% nucleotide identity to *Bathycoccus prasinos* BLA77). Results for 16S rDNA clone sequences were less clear and an unknown endosymbiont or other material may have been present (Table S1). Therefore, only scaffolds with ≥ half of predicted ORFs with best BLASTp hits to predicted protein sequences in other Mamiellales genomes (i.e. *Micromonas* and *Ostreococcus*), that fit other criteria as well (see *Experimental procedures*), were considered derived from the *Bathycoccus* nuclear genome and used in subsequent analyses. Two distinct G + C signatures were observed in the *Bathycoccus* nuclear metagenome similar to what has been seen in whole genome sequences from cultured members of the Mamiellales. The ‘anomalously low-G + C’ region of 38% G + C was about 10% lower than the overall metagenome average of 48%. These regions of considerably lower G + C content were first observed in *O. tauri* (58% for the whole genome and 52% for the low G + C region) and *Micromonas sp.* RCC299 (64% and 50% respectively) and have been hypothesized to serve as a sex chromosome that encodes convergent, overlapping genes (Derelle *et al*., 2006; Worden *et al*., 2009). The specificity of recovered ORFs, as well as detection of low G + C scaffolds with a differential from the overall average within range of that observed in *Ostreococcus* and *Micromonas*, indicating that the assembled and filtered metagenome is specific to the *Bathycoccus* nuclear genome.

**Table 2:** Summary of water samples from which metagenomes were sequenced and corresponding metadata

Site	Date (d/m/y)	Location (lat.; long.)	Depth (m)	T (°C)	S (ppt)	PO_4_ (µM)	NO_3_ (µM)	NH_4_ (nM)	Chl *a* (mg m^−3^)
*Atlantic*									
Sta. 13 Sort	12/07/06	12.378; −27.241	64	21.76	35.89	0.04	0.20	na	na
*Pacific*									
H3	10/10/07	36.740; −122.020	5	12.28	33.47	1.121	8.860	na	4.1966
67–70	09/10/07	36.129; −123.490	10	15.57	33.12	0.609	0.511	na	2.7156
67–155	07/10/07	33.286; −129.428	5	19.02	33.19	0.655	0.013	15	0.0998
67–155	06/10/07	33.286; −129.428	86	13.39	33.13	0.579	0.397	19	0.9398

For Atlantic samples reported NO_3_ measurements reflect NO_3_ + NO_2_.

T, temperature; S, salinity.

A *pho4* gene was identified in the *Bathycoccus* metagenome, with highest amino-acid sequence similarity to that of *Ostreococcus* (65%, *O. lucimarinus* XP_001422167). Similarities between *pho4* genes from these Mamiellales and those in *Micromonas* were low (*Bathycoccus* had 29% similarity to *Micromonas* RCC299 ACO69637 and 27% to *M.* CCMP1545 XP_003062965). Surprisingly, the similarities between *pho4* from *Ostreococcus* or *Bathycoccus* were lower to *Micromonas* than to *Tetraselmis chui* (50% similarity with *Bathycoccus*), a more distant prasinophyte. *Bathycoccus* and *Ostreococcus* also appeared more closely related to each other than to *Micromonas* based on 18S rDNA analyses (Fig. S2), see also (Guillou *et al*., 2004; Worden, 2006).

**Fig. 2:**
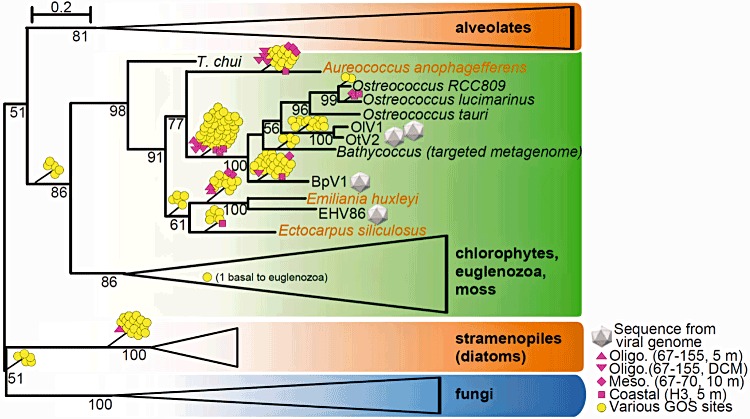
Phylogeny of eukaryote and eukaryotic virus *pho4* protein sequences, including the *pho4* gene from the *Bathycoccus* targeted metagenome. While most prasinophyte gene sequences branched together in the ‘green-clade’, the *Micromonas pho4* homologues were phylogenetically distant and not close to this region of the eukaryotic *pho4* gene tree. *Pho4* sequences retrieved from traditional metagenomes (fuchsia and yellow bubbles) were assigned to branches on this reference tree using pplacer. This maximum-likelihood tree was reconstructed using phyML and the WAG matrix. Bootstrap values represent percentage of 100 replicates. Only metagenomic reads mapped with support (*P* ≥ 0.75) are shown. For balance in taxon sampling only one (EGB10629) of three (EGB10629, EGB08825, EGB12603) *Aureococcus pho4* gene sequences that branched within this region of the tree with statistical support in a preliminary phylogenetic reconstruction (not shown) was included. These three versions ranged from 66% to 72% similarity at the amino-acid level.

Recovery of *pho4* genes from host genera and other eukaryotes allowed us to construct a robust phylogeny for the corresponding viral and host *pho4* protein sequences. BpV-1, OlV-1 and OtV-2 *pho4* sequences branched with those of *Bathycoccus* and *Ostreococcus* while the EhV-86 *pho4* gene branched with its host *E. huxleyi* (Fig. 2). The analysis suggests that multiple horizontal gene transfer (HGT) events occurred – although the direction of those events is still unclear (whether from host to virus, or virus to host). Phylogenetic analysis supports the hypothesis that the EhV-86 and *Ostreococcus* viral *pho4* genes were derived from their respective host genera, rather than a common viral ancestor. Still, other gene exchange scenarios are possible. For instance, the exchange could have occurred from host to virus, virus to virus and then virus to another host lineage, explaining the presence of ‘green’-like *pho4* genes in, e.g. the haptophytes. The pattern of multiple exchanges, resulting in an indirect virally mediated exchange of host genes to another host has been hypothesized for the photosynthetic reaction centre genes *psbA* and *psbD* in cyanobacteria and cyanophages (Sullivan *et al*., 2006). Two *pho4* genes from *Ostreococcus* viruses branched together and have higher similarity (across all positions) to one another than to homologues from *Ostreococcus* itself. A *pho4* sequence is not available from *Ostreococcus* RCC393, the host strain against which OtV-2 was isolated, although a sequence from another *Ostreococcus* Clade OII member (*Ostreococcus* RCC809) was included. The fact that OlV-1 and OtV-2 sequences branched together could indicate they originated from a common ancestor rather than from their respective hosts. Without an OtV-2 host-encoded *pho4* sequence in the tree it is not possible to determine if placement of the OtV-2 version would differ if the host sequence was included. Finally, host cross-infectivity levels of Phycodnaviridae family members are not well known and could influence acquisition and retention patterns.

Horizontal gene transfer events similar to those hypothesized here for the *pho4* gene in marine systems have been reported for *E. huxleyi* and EhV-86 sphingolipid biosynthesis genes. Sphingolipid biosynthesis genes were hypothesized to have been acquired by the virus from its host (Monier *et al*., 2009) and viral glycosphingolipid molecules have been shown to be synthesized by the haptophyte during infection (Vardi *et al*., 2009). These molecules have been detected in the natural environment and are possibly related to bloom dynamics of the host (Vardi *et al*., 2009). In contrast to the broad distribution of *pho4* genes in viruses that each infect different marine eukaryotic taxa or lineages, the hypothesized sphingolipid pathway HGT has so far only been reported between *E. huxleyi* and EhV. In addition to the *pho4* homologues present in various algae and their viruses, we found a close homologue of the BpV-1 and BpV-2 encoded Cation Channel-forming *hsp70* family gene in the *Bathycoccus* metagenome (scaffold C503; this gene is also present in *O. lucimarinus* and *Micromonas* RCC299), suggesting this gene may have been acquired through HGT and is sufficiently advantageous to be retained by the recipient genome. Homology has been reported between 11 predicted genes in OtV-1 and host-encoded (*O. tauri*) genes (Weynberg *et al*., 2009). Studies exploring the expression of virally encoded *pho4* genes and other putatively exchanged genes should enhance our understanding of viral impacts on oceanic phytoplankton as well as environmental factors controlling viral proliferation.

### Phylogenetic relationships between algal *pho4* genes and implications for function

The phylogenetic relationships of phytoplankton *pho4* homologues inferred from the protein sequences (Fig. 2) were inconsistent with ‘species phylogenies’ based on 18S rDNA and chloroplast gene trees (Andersen, 2004; Keeling *et al*., 2005; Worden and Not, 2008). In our analysis, a ‘green-clade’ consisting of *pho4* homologues from the prasinophytes *Bathycoccus*, *Ostreococcus* and *T. chui* formed a sister group to other members of the Plantae, especially green algae. However, this ‘green-clade’ also included *pho4* homologues from stramenopiles (*E. siliculosus* and *A. anophagefferens*) and the haptophyte *E. huxleyi*. High representation of green algal or plant-like genes in haptophytes has been reported previously (Hampl *et al*., 2009; Cuvelier *et al*., 2010). Interestingly, *E. siliculosus* appeared to only have the ‘green’*pho4* gene-version and *Aureococcus* (Gobler *et al*., 2011) had multiple versions, three of which were ‘green’-like (Fig. 2). In contrast, *pho4* genes from the diatoms *Thalassiosira pseudonana* and *Phaeodactylum tricornutum* (Armbrust *et al*., 2004; Bowler *et al*., 2008) grouped together in a position outside of the ‘green-clade’, as would be expected based on their evolutionary history inferred by chloroplast gene trees. Anomalous phylogenetic patterns have also been reported for phytoplankton ammonium transporters (McDonald *et al*., 2010). However, in most cases, each different picoprasinophyte AMT type (i.e. forming distinct clades within the AMT-gene tree) was present in all of the sequenced picoprasinophyte genomes. In contrast, despite the presence of ‘green’-like PHO4 superfamily members in disparate lineages, this version was not found in the picoprasinophyte *Micromonas*; their *pho4* genes branched in an unsupported position basal to metazoans (not shown). There are several possible explanations for the observed phylogenetic distribution of *pho4* genes in eukaryotic algae. The fact that the stramenopile and haptophyte *pho4* homologues grouped with green algae may have been influenced by taxon undersampling, particularly the lack of available homologues from red algae apart from *C. merolae*. However, it may also reflect ancestral characteristics with differential loss or divergence in some taxa (e.g. diatoms) or gain from a proposed cryptic green algal endosymbiont in the chromalveolates (Moustafa *et al*., 2009). Alternatively, HGT events may have occurred between distantly related organisms, possibly involving virally mediated exchange, leading to the observed phylogenetic relationships. HGT events have been reported between distant eukaryotic organisms, e.g. carotenoid production related genes in fungi and aphids (Moran and Jarvik, 2010).

All of the *pho4* genes within the ‘green-clade’ that have been experimentally characterized serve as high-affinity transporters. The *T. chui* encoded protein (AAO47330) acts as a high-affinity PO_4_ permease and the gene is specifically upregulated in P-deplete conditions (Chung *et al*., 2003). Its branching position was basal to *pho4* genes from several of the algae and viruses analysed, suggesting it represents a more ancestral version of the gene (Fig. 2); it showed a high degree of similarity to the algae at terminal nodes of the ‘green-clade’. The *E. huxleyi pho4* gene is expressed under PO_4_ depletion (Dyhrman *et al*., 2006a) and similar results have been reported for an *Aureococcus* version (Wurch *et al*., 2011), both of which are embedded within this clade (Fig. 2). Thus, we hypothesize that the other algal *pho4* genes branching within this region of the tree are also high-affinity PO_4_ transporters. Notably, in the *Ostreococcus* viruses, a 54-amino-acid segment was missing from the *pho4* gene that is present in host versions. In the same region 19 amino acids were absent from BpV-1 that were present in the host version. How these deletions affect function is still unclear; deletions are seen in this region in some eukaryotic taxa as well, although involving fewer amino acids.

Most of the eukaryotic viral genomes analysed herein also encoded the gene *phoH* (Table 1). Although not a transporter this is relevant because *phoH* is found in the PHO (phosphate) regulon of *E. coli* and other bacteria, although its precise role is unclear (see discussions in Martiny *et al*., 2006; Sullivan *et al*., 2010). We identified *phoH* homologues in all available Mamiellales-virus genomes (OtV-1, OtV-2, OtV-5, OlV-1, BpV1, BpV-2 and MpV-1) but not EhV-86 (Table 1). Unlike the *phoH* genes in cyanobacteria and cyanophages (present in both hosts and viruses) or *pstS* and *pho4* genes, which were found in all sequenced hosts, *phoH* genes appeared to be present only in the *O. tauri* and *E. huxleyi* genomes, based on BLASTp and tBLASTn similarity searches against available proteomes and genomes. In addition, the *O. tauri*-encoded version did not appear to be closely related to versions in viruses infecting *Ostreococcus*.

### PHO4 superfamily members in the natural environment

**Fig. 3:**
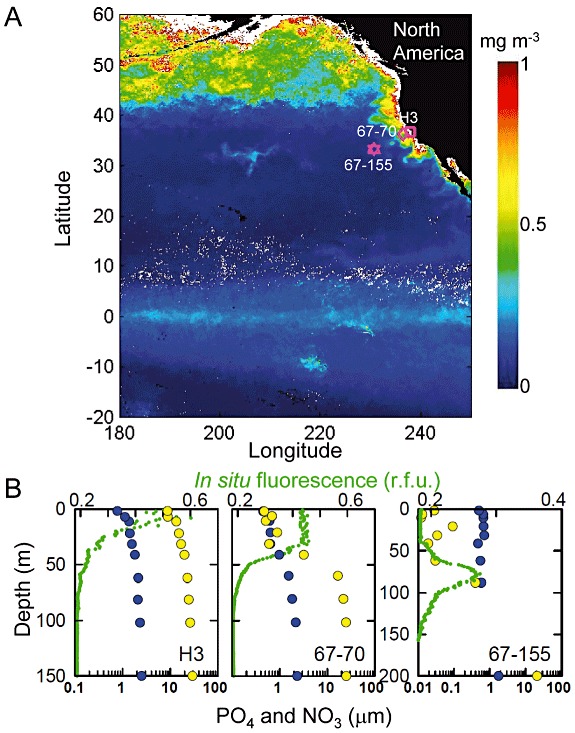
A. Sample sites for Pacific Ocean metagenomes and satellite inferred chlorophyll a concentrations (mg m^−3^) over a period spanning the cruise (14 September–8 November 2007). Black indicates land, white missing data, pink symbols indicate sample sites. B. Phosphate (blue) and nitrate (yellow) concentrations as well as *in vivo* fluorescence (green) at sample collection sites.

**Fig. 4:**
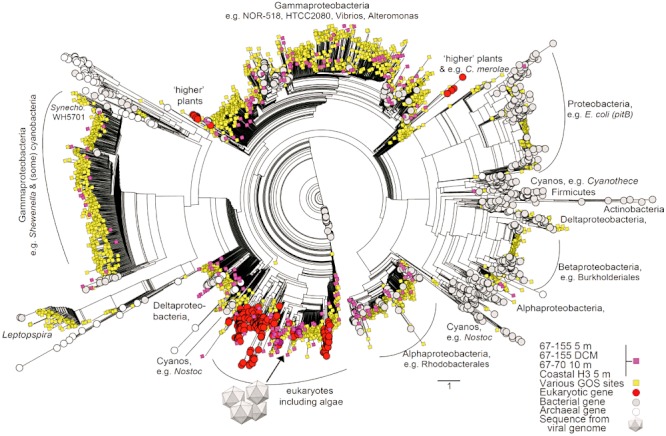
Metagenomic sequences assigned to branches on a maximum-likelihood tree built from the PHO4 Pfam alignment. Squares represent (yellow) GOS sequences and (fuchsia) metagenomic sequences containing the PHO4 Pfam model (see *Experimental procedures*) from our Pacific Ocean transect (Fig. 3). Two hundred and forty and 2751 putative PHO4 sequences were detected in the Pacific metagenomes and GOS data respectively using our HMM. Due to the number of sequences in the Pfam alignment and computational time needed, bootstrap analysis was not performed for the maximum-likelihood reference tree.

**Fig. 5:**
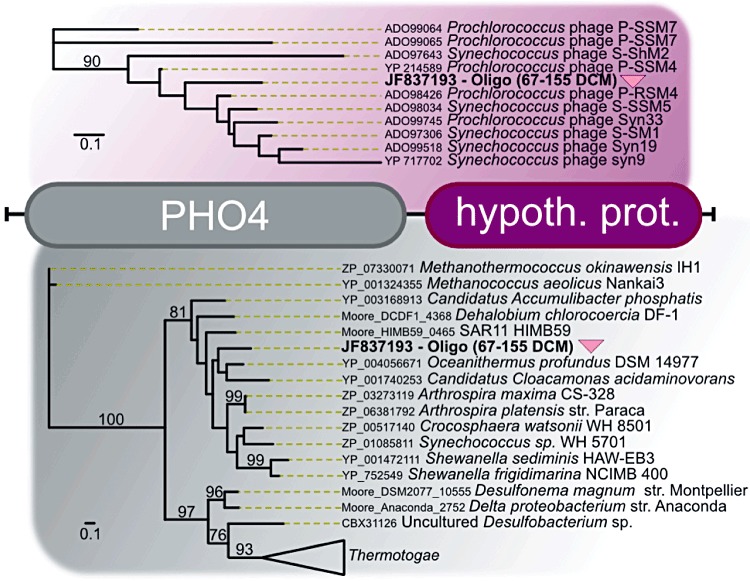
Architecture of a cloned Pacific Ocean metagenome sequence and phylogeny of detected ORFs. This cloned sequence (1139 nt, JF837193) was retrieved from the DCM at Pacific Ocean Station 67–155. Maximum-likelihood phylogenetic trees were reconstructed with phyML using JTT matrix and 100 bootstrap replicates. Homologous sequences were retrieved by BLASTp searches against NCBI-NR and Moore marine microbial genomics databases. Note that taxon sampling for the hypothetical protein phylogeny is influenced by the fact that most sequenced marine phage genomes are from cyanophages.

We scanned metagenomes from three Pacific Ocean environments (Fig. 3), coastal (high PO_4_), mesotrophic (moderate PO_4_) and oligotrophic (moderate PO_4_ and low nitrogen) for members of the PHO4 superfamily (Table S2a) as well as Global Ocean Sampling (GOS) data (Rusch *et al*., 2007) using a HMM (Fig. S3, Table S2b). The detected PHO4 sequences were assigned to branches on a fixed maximum-likelihood reference tree built from the overall Pfam alignment (Fig. 4). A large fraction of environmental sequences were assigned to two groups containing Gammaproteobacteria. One of these groups contained sequences from cultured *Shewanella* strains and some cyanobacterial gene sequences (*Arthrospira* and *Synechococcus* WH5701) branched in the same region of the tree. WH5701 is a halotolerant strain and some environmental sequences assigned to this group might be from the nitrogen fixing cyanobacterium *Crocosphaera*. The *Crocosphaera* gene model containing the PHO4 domain (ZP_00517140) appeared to be missing a section of the conserved C-terminal domain that is part of the PHO4 Pfam model and was therefore not included in the tree, but had high similarity to the WH5701 version (ZP_01085811; 69% identity at the amino-acid level). *Prochlorococcus* and *Synechococcus* do not appear to encode genes containing the PHO4 Pfam domain. Environmental sequences were assigned to other bacterial groups as well (Fig. 4). The PHO4 Pfam model is composed of both high- and some low-affinity transporters and affinities could not be inferred based on placement in the overall Pfam analysis (Fig. 4). Although there appear to be numerous bacterial *pho4* homologues in environmental community DNA (Fig. 4), if bacteriophages exchange this gene with their hosts, as eukaryotic viruses appear to, then any number might belong to phage rather than bacteria. Indeed, the flanking region of a Sanger sequenced shotgun clone from the Pacific Ocean deep chlorophyll maximum (DCM, 86 m) metagenome, recognized by our PHO4 superfamily HMM, indicated it was phage derived. In our phylogenetic analysis of this clone, the *pho4*-encoding ORF branched in an unsupported position with several bacterial taxa, close to the marine alphaproteobacterium strain HIMB59 (Fig. 5). In contrast, the flanking ORF in this cloned sequence encoded a hypothetical protein so far only seen in cyanophages.

Three hundred and nineteen environmental sequences were assigned to branches within the region of the PHO4 superfamily tree containing cultured eukaryotic algae, the *Bathycoccus* targeted metagenome *pho4* gene sequence and the viral versions discussed above (Fig. 4). These environmental sequences were then assigned placements on the refined-eukaryotic *pho4* tree (Fig. 2), which included more positions than the superfamily tree. The majority of eukaryotic environmental sequences grouped with prasinophytes, stramenopiles, haptophytes or corresponding viruses. A large number of those assigned to the Mamiellales (*Bathycoccus*/*Ostreococcus*) clade appeared to be from viral DNA templates (i.e. those assigned to OlV-1/OtV-2 or BpV-1) rather than host-derived templates. For the haptophyte lineage, environmental sequences were not placed with *E. huxleyi* or its virus EhV-86, but rather branched in a position basal to the *E. huxleyi*-EhV-86 node, suggesting they may have originated from other prymnesiophyte taxa, which include widespread uncultured marine groups (Worden and Not, 2008; Liu *et al*., 2009; Shi *et al*., 2009; Cuvelier *et al*., 2010), or potentially their viruses. Sequenced genomes are not available for *Pelagomonas* (or *Pelagomonas*-infecting viruses); therefore, a *pho4* gene from this taxon (assuming it contains the *pho4* gene) was not included in our analysis. Several *Pelagomonas*, but not *Aureococcus*, SSU rDNA sequences were present in clone libraries (data not shown) from the Pacific sites (Table 2). Hence, environmental *pho4* sequences assigned to the *Aureococcus* node were likely from its more oceanic relative *Pelagomonas*. The analysis showed that eukaryotic algae and viruses inhabiting both PO_4_ replete (Fig. 3) and deplete (Figs. 1 and S3, and some cultured taxon isolation sites) environments contained the putatively high-affinity inorganic PO_4_ transporter gene (Fig. 2). Given the high degree of homology between host and respective viral *pho4* sequences, unambiguous assignment to either host- or infecting virus branches was not always possible, particularly for short-metagenomic reads with limited phylogenetic information. The fact that our Pacific Ocean metagenome sequences came from larger size fractions (0.1 to ≤ 0.8, 0.8 to ≤ 3.0 and 3.0 to ≤ 20 µm) as opposed to sampling protocols that target the viral size fraction (e.g. tangential flow filtration for < 0.1 µm sized particles), indicates that the virally derived environmental sequences were present in infected hosts, especially for the two largest size fractions we sequenced. The proportion of viruses in nature that encode the *pho4* gene is still not known.

### Broader implications

In marine environments, P-availability is linked to primary production levels and shifts in phytoplankton community composition. PO_4_-uptake via different molecular mechanisms mitigates P-starvation and is important for survival in oligotrophic waters (e.g. Dyhrman *et al*., 2006b; Coleman and Chisholm, 2010). The distribution of the ‘green’-like *pho4* homologues across green algae, haptophytes and some stramenopiles, indicates this putatively high-affinity transporter is important to physiological success. Together with previously hypothesized *pstS* HGT from cyanobacteria to cyanophages (Sullivan *et al*., 2010), the host-virus exchanges hypothesized herein show unexpected commonalities in the retention of PO_4_-uptake related genes in viral genomes from both viruses that infect eukaryotic hosts and bacteriophages.

Experimentally, *E. huxleyi* viral lysis rates are reportedly higher in P-replete than P-deplete conditions (Bratbak *et al*., 1993). Similar observations were reported for *Synechococcus* grown in P-deplete conditions, in which they appear to be infected and lysed at significantly lower rates by the bacteriophage S-PM2, than in P-replete conditions (Wilson *et al*., 1996). The presence or absence of PO_4_-uptake related genes in the genome of the infecting virus might mechanistically underpin these observations. For example, the S-PM2 genome does not appear to encode *pstS* (Mann *et al*., 2005), consistent with its low lysis rates in P-deplete conditions. Viral nutrient requirements and the ability of a virus, or lack thereof, to increase host nutrient uptake under limiting conditions could explain many such results. Moreover, presence of PO_4_-uptake related genes in a significant fraction of sequenced viral genomes, but not known nitrogen-uptake related genes, supports the hypothesis that viruses are more susceptible to PO_4_-limitation than nitrogen limitation due to a high nucleic acid to protein ratio. The prevalence of *pho4* in marine viral genomes indicates it may alleviate bottom-up control of viruses by PO_4_-limitation, allowing them to propagate even under low PO_4_ conditions. This in turn could influence phytoplankton top-down controls, enhancing the ability of eukaryotic viruses and bacteriophages alike to replicate and induce host mortality. Thus, expression and manipulation of PO_4_-uptake related genes presumably influence both the fitness and demise of multiple marine microbial taxa. Our data reveal evolutionary pressures felt by marine viruses and highlight the complex influences of nutrient bioavailability on microbial interactions and dynamics.

## Experimental procedures

### Identification of transporter genes in virus genomes

Transporter protein sequences and corresponding annotations were retrieved from TransportDB (Ren *et al*., 2007). To avoid biases resulting from different gene prediction methods we predicted ORFs for all the eukaryotic viral genomes investigated (Table 1). Viral genome sequences were downloaded from GenBank, and stop-stop ORFs with a minimum size of 60-amino-acid residues were retrieved. A combination of BLASTx and BLASTp searches against TransportDB were performed and BLAST hits having an *e*-value less than 0.001 were considered for further analysis. Results were controlled using BLASTp searches against NR and searches against Pfam-A using the hmmscan module of HMMer 3 (Eddy, 2009).

### Environmental sample collection and contextual analyses

Samples were collected using a rosette equipped with Niskin bottles, a CTD and fluorometer on two research expeditions, one aboard the *R/V* Seward Johnson (July 2006) and the other on *R/V* Western Flyer (October 2007). The first cruise was in the tropical Atlantic Ocean and the sample sequenced here was collected on 12 July 2006 at Station 13 (SJ0609) from the deep chlorophyll maximum (Fig. 1, Table 2). One L of water was filtered by gravity onto a 0.45 µm pore size Supor filter until reduced (concentrated) to a volume of 10 ml, this concentrated sample was then used for flow sorting as below. The second cruise was performed in the eastern North Pacific and samples collected at three sites (Table 2). In both cases samples for phosphate and nitrate analysis were collected at multiple depths from the surface to the base of the euphotic zone. For DNA for traditional bulk metagenomic sequencing, approximately 200 l of water were filtered first through a 20 µm mesh, and then sequentially through 293 mm diameter, 3 µm pore size filters (Pall Sciences Versapor-3000T), a 0.8 µm pore size filters (Pall Sciences Supor-800) and finally a 0.1 µm pore size filters (Pall Sciences Supor-100). Surface ocean chlorophyll concentrations (Figs 1 and 3; Fig. S3) were derived from the MODIS instrument carried on NASA's Aqua satellite (http://oceancolor.gsfc.nasa.gov).

### Metagenome templates and construction

Processing for materials used to construct the targeted metagenome (sorted population) and the traditional metagenomes (size fractionated filters) was different. For the former, a 454-FLX and Sanger blended metagenomic assembly was generated for *Bathycoccus*. The analysed cells were first sorted by flow cytometry based on distinctive scatter and chlorophyll signals (Fig. S1) using an InFlux cell sorter (BD), directly after water collection. To avoid contamination, the instrument was cleaned extensively. Sheath fluid was 0.2 µm filtered, autoclaved PBS made in 18.2 MegaOhm H_2_O and 750 ml were run through the instrument after cleaning (immediately before sorting). Contamination control materials were from left and right test deflections run for 1 min (sample flow rate ∼25 µl min^−1^). All sort droplets were immediately frozen cryogenically. Upon return to land the population sort was resorted to enhance purity levels (Fig. S1). The sample and controls were then amplified by MDA and the products debranched and precipitated prior to sequencing according to methods in Cuvelier and colleagues (2010). Universal 16S rRNA gene primers were used to verify the absence of product in controls (indicating the instrument provided no prokaryotic contamination). A combination of Sanger shotgun sequencing and 454 pyrosequencing was then performed on the DNA as detailed in Cuvelier and colleagues (2010). For Sanger sequencing, 3 Kbp shotgun libraries were constructed using debranched MDA products and end-sequencing yielded 71 328 reads (totalling 52.83 Mbp). Pyrosequencing was also performed on debranched MDA products using the Genome Sequencer FLX System (454 Life Sciences) according to the manufacturer protocol and resulted in 118 Mbp of sequence. Pyrosequence reads were assembled using the 454 Newbler assembler version 1.1.02.15 and the consensus sequence shredded into 1 Kbp shreds with 100 bp overlaps. The 454 shred data were assembled with Sanger sequences using the lucy trimmer, to remove vector and low-quality sequence, and the Paracel Genome Assembler (lucy version 1.19p, PGA version 2.6.2). The assembly process generated 1929 contigs containing 9 859 508 bp.

To assess purity of the *Bathycoccus* sort we constructed 18S rDNA clone libraries using universal 18S rDNA primers (Moon-van der Staay *et al*., 2000). The resulting 741 sequences were analysed using Mothur v1.17 (Schloss *et al*., 2009). Sequences were first aligned against the SILVA 18S rRNA gene reference alignment (Pruesse *et al*., 2007). After end-trimming, clustering at 99% sequence identity resulted in six clusters and representatives of these were used for phylogenetic analysis. The maximum-likelihood tree was constructed using phyML v2.4 (Guindon and Gascuel, 2003) using an alignment where all gap-containing positions were removed and the TrNG substitution model was selected by AIC criterion (MrAIC script, distributed by J.A.A. Nylander, Uppsala University), and 100 bootstrap replicates. Because mitochondrial 16S rDNA databases are limited, BLASTn was performed against two databases, NCBI reference genomes and NCBI non-redundant nucleotide database (NT) using the 16S rDNA clones generated herein as query sequences.

In order to further insure that only scaffolds originating from *Bathycoccus* were analysed, ORFs spanning at least 180 nucleotides (between two stop codons) were retrieved using EMBOSS GetORF (Rice *et al*., 2000). The resulting putative ORFs were used as BLASTp queries (Altschul *et al*., 1997) against the NCBI non-redundant database (NR-DB). Taxonomic information for each of the best BLASTp hit (*E*-value cutoff: 1e^−3^) was retrieved using the NCBI taxonomy database and assigned to the corresponding ORF. A scaffold was classified as *Bathycoccus*-like if more than half of its predicted ORFs had their best BLASTp hits (*E*-value cutoff: 1e^−3^) against a protein sequence originating from any of the five available prasinophyte genomes (Derelle *et al*., 2006; Palenik *et al*., 2007; Worden *et al*., 2009), including the publically available but unpublished genome from *Ostreococcus* RCC809. That is, at least three ORFs had to be detected on the scaffold, and two or more of these had to have best BLASTp hits to prasinophytes in order to be included in subsequent analyses. Additionally, scaffolds smaller than 7 kb were discarded. Of 10 Mb total assembly, scaffolds composing 7.1 Mb fit the criteria of more than half the ORFs being prasinophyte-like (with a minimum of three predicted ORFs), and among these 5.1 Mb were scaffolds of 7 kb or more (183 scaffolds).

For each Pacific Ocean traditional metagenome, three different size fractions were extracted and sequenced independently. The nominal size fractions were 0.1 to < 0.8 µm, 0.8 to < 3 µm and 3 to < 20 µm. DNA was extracted from a fragment of each 293 mm filter using a sucrose protocol as described in Cuvelier and colleagues (2010). These materials were then divided and sequenced using the 454-FLX platform and Sanger sequencing.

### PHO4 homologue searches

The PHO4 model alignment from Pfam (PF01384) was retrieved and used to build a hidden Markov (HMM) model based on amino-acid sequences using hmmbuild [part of HMMer 3 (Eddy, 2009)]. This HMM was then used with hmmsearch (also part of HMMer 3) to detect PHO4 homologues not yet integrated in the PF01384 model alignment. Searches were conducted against microbial eukaryotic proteomes not yet published or released in NR-DB and Uniprot (Apweiler *et al*., 2010) but publicly available (i.e. *E. huxleyi*, *A. anophagefferens*, *Ostreococcus sp*. RCC809) and NR-DB, allowing the detection of *E. siliculosus* (Cock *et al*., 2010), *Chlorella sp*. NC64A and *Volvox carteri f. nagariensis* homologues. The detected putative PHO4 sequences were then manually validated using BLASTp searches against NR-DB. It should be noted that the gene name *pho4* has also been used to refer to genes that are not directly related to PO_4_ transport.

Environmental PHO4 sequences were identified from GOS [ORFs were retrieved from CAMERA (Seshadri *et al*., 2007)] and CN207 [after stop-stop ORF predictions with a minimum size of 40 amino-acid residues, as implemented by ORF_finder, part of the RAMMCAP analysis pipeline (Li, 2009)] datasets using the PF01384 HMM enriched with other detected PHO4 sequences (added to the model alignment with hmmalign) using hmmsearch with an *E*-value threshold of 1e^−10^ and gathering cutoff. The percentage of *pho4* genes detected in our 454-FLX reads was lower than from GOS samples (Sanger sequences) or some of our Sanger libraries (Table S2). Frequency differences for PHO4 superfamily members in different samples were not compared since they could be a function of (i) the two data types (Sanger and 454-FLX) used or (ii) the fact that short reads encode less information for successful domain detection. Furthermore, differences in nominal pore sizes, samples and relative depth of sequencing compound difficulties in interpreting frequency results.

### Phylogenetic analyses

The overall PHO4 superfamily phylogenetic tree reconstruction (Fig. 4) was based on a non-redundant Pfam model alignment PF01384 with the addition of newly identified PHO4 amino-acid sequences (see above paragraph), which were aligned using hmmalign (HMMer 3). To reduce the number of taxa in the alignment prior to phylogenetic analysis representative sequences were selected from a 95% similarity clustering using Uclust (Edgar, 2010). In addition, short sequences (i.e. smaller than 200-amino-acid residues) were removed from the multiple sequence alignment. The final alignment was composed of 954 protein sequences. The maximum-likelihood PHO4 tree was reconstructed using RAxML 7.0.4 (Stamatakis, 2006), an estimate gamma shape parameter, a WAG matrix and empirical amino-acid frequencies. Statistical support was not computed for this reference tree due to the computational time needed with the large number of sequences and positions in the Pfam alignment, as well as the use of maximum-likelihood methods.

From the overall PHO4 superfamily protein tree, a region of the tree where most eukaryotic algal PHO4 protein sequences branched was identified and corresponding eukaryotic sequences were extracted from the alignment. This allowed us to construct an alignment that retained more informative positions than the entire Pfam alignment. Sequences were aligned using clustal W2 and the alignment was curated manually. A few additional sequences were added using T_Coffee v. 8.91 (Notredame *et al*., 2000). The majority of gap-containing sites were removed prior tophylogenetic analysis. This alignment served for a maximum-likelihood phylogenetic reconstruction using phyML v. 3 (Guindon and Gascuel, 2003) using Jones-Taylor-Thornton (JTT) matrix and 100 bootstrap replicates.

Homologues to the hypothetical protein and PHO4 superfamily protein sequences encoded on a potential marine bacteriophage metagenomic Sanger-sequenced shotgun clone were retrieved using a combination of BLASTp and tBLASTn searches against NR-DB and NCBI reference genomes and genomes of the Moore Marine Microbial Genomics Initiative. Sequences were aligned using T_Coffee v.8.91. Resulting multiple sequence alignments were cleaned using Gblocks (Castresana, 2000) allowing 50% of gapped-positions and was manually inspected using seaview 4 (Gouy *et al*., 2010). Phylogenetic reconstructions were conducted using phyML v. 3 with JTT matrix and 100 bootstrap replicates.

### Placement of metagenomic PHO4 superfamily sequences

PHO4 superfamily members detected in traditional metagenomes from the Pacific (as well as in GOS data) using our HMM search were then assigned to branches or nodes on the PHO4 protein family tree using pplacer (Matsen *et al*., 2010). A WAG matrix was used and Bayesian posterior probabilities computed to establish statistical support of the placements. The resulting tree with mapped environmental sequences was visualized and edited with Dendroscope (Huson *et al*., 2007) and taxonomic information for each of the tree node was retrieved through UniProtKB identifiers (Apweiler *et al*., 2010). Environmental sequences assigned to the eukaryotic/algal region of the superfamily tree (Fig. 4) were extracted and used for a second round of phylogenetic placement (with same parameters as the initial run) on the manually curated eukaryotic PHO4 phylogenetic tree. Only environmental PHO4 sequences assigned placements that retained statistical support (i.e. *P* ≥ 0.75) were considered further.

### Nucleotide sequence accession numbers

Traditional metagenome Sanger sequences from this study have been deposited in GenBank under Accession Numbers JF837193-JF837211 and 454-FLX sequences are in the CAMERA database (https://portal.camera.calit2.net/gridsphere/gridsphere). The targeted *Bathycoccus* metagenome was deposited in NCBI genomes under accession AFUW00000000.

## Supporting information

**Fig. S1.** Flow cytometry histograms of the pre-concentrated tropical Atlantic sample that was sorted. The arrows indicate the target population. Top panels are the sample as it appeared at sea during sorting. The right hand panel shows *Synechococcus* cells (R1), which have been gated out from the left hand panel. The bottom panel shows the sorted population as thawed and rerun on land where it was resorted for purity. R1 again shows the position where *Synechococcus* would be expected. Note cytometer set up is never identical between runs (even if strived for) hence position of populations in top and bottom panels is slightly different.

**Fig. S2.** Analysis of an 18S rDNA clone library from the sorted, MDA amplified population template. All of the 741 successfully sequenced 18S rDNA clones were phylogenetically placed with *Bathycoccus*. Prior to this maximum-likelihood analysis (PhyML) sequences were clustered at 99% identity (sequences were not manually curated) and a single representative of each of the six resulting clusters used in the alignment and tree. Eighthundred and thirty homologous positions were analysed after gap removal with 100 boot straps. After discarding gapped positions and ambiguous positions in the alignment, differences between *Bathyoccus* sequences were so few that it resulted in the observed polytomy in this reconstruction.

**Fig. S3.** Aqua MODIS chlorophyll concentration 1 February 2004–30 September 2005, the period spanning collection of sequenced GOS samples. Grey indicates land, white missing data, yellow circles indicate sites at which PHO4 was detected and black circles indicate those where no PHO4 were detected although the metagenome from the site was analysed.

**Table S1.** BLASTn results of cluster representatives from the population sort 16S rDNA clone library. The sequences appear to be distantly related to those in both NCBI environmental nucleotide (env_nt) and reference genomic sequence (ref_genomes) databases and most related to uncultured bacteria from the nucleotide non-redundant database (nt). Note clone library sequence assemblies were not manually curated.

**Table S2a.** Number of metagenome sequences and number of identified PHO4 sequences. Those under Total Pho4 were placed on the PHO4 tree (after retrieval using the HMM), but do not necessarily have statistical support for their respective placements. Those under Euk/Virus heading could result from hosts or their respective viruses, while those in the viral column were unambiguously assigned to viruses. For both these categories only sequences retaining placement support (with probability ≥ 0.75) are included. Total non-redundant ORFs are based on six-frame translation and minimum length of 40 and 60 amino acids for 454-FLX and Sanger respectively. Breakdown of read numbers is given for all sequencing technologies in Table S4.

**Table S2b.** Summary of PHO4 detected in the Global Ocean Sampling predicted protein datasets as deposited in CAMERA. All PHO4 were identified using the HMM and those in the columns Euk/Virus or Virus also had supported positions in the phylogenetic analysis (with probability ≥ 0.75). Sequences in Euk/Virus could result from hosts or their respective viruses, while those in the viral column were unambiguously assigned to viruses. Total reads does not reflected the number of predicted proteins and the number was not easily retrievable.
