# Supplementary material for: Phosphate transporters in marine phytoplankton and their viruses: cross-domain commonalities in viral-host gene exchanges
Source: Environ Microbiol. 2012 Jan;14(1):162–76. doi: 10.1111/j.1462-2920.2011.02576.x (PMC3429862; doi:10.1111/j.1462-2920.2011.02576.x)
Supplement: Supplementary file 1 [file emi0014-0162-SD1.pdf]

Supplementary Tables

Table S1. BLASTn results of cluster representatives from the population sort 16S rDNA clone library. The sequences appear to be distantly related to those in both NCBI environmental nucleotide (env_nt) and reference genomic sequence (ref_genomes) databases and most related to uncultured bacteria from the nucleotide non-redundant database (nt). Note clone library sequence assemblies were not manually curated.

16S clust[a]	best BLASTn HSP[b]	nt	env_nt	ref-genomes
**#6**	**Accession**	HQ672230	AACY023936572	NC_006832
	**Descrip.**	Uncultured bacterium clone F9P2610_S_P18	Marine metagenome	*Ehrlichia ruminantium*
	**Score**	1070	366	459
	**% ID**	94% (663/704)	77% (562/731)	79% 576/728)
**#19** (#21, #2)	**Accession**	HQ672230	AACY023245853	NC_010793.1
	**Descrip.**	Uncultured bacterium clone F9P2610_S_P18	Marine metagenome	*Orientia tsutsugamushi*
	**Score**	2010	593	584
	**% ID**	93% (1278/1370)	77% (924/1205)	77% (930/1213)
**#14** (#10)	**Accession**	HQ672230	AACY023245853	NC_010793
	**Descrip.**	Uncultured bacterium clone F9P2610_S_P18	Marine metagenome	*Orientia tsutsugamushi*
	**Score**	2032	562	580
	**% ID**	94% (1282/1370)	76% (920/1207)	77% (929/1213)
**#5**	**Accession**	HQ672230	AACY020472379	NC_007685
	**Descrip.**	Uncultured bacterium clone F9P2610_S_P18	Marine metagenome	*Dictyota dichotoma*
	**Score**	1522	448	460
	**% ID**	93% (982/1058)	76% (741/973)	77% (739/963)
**#11** (**#**15, #3, #17, #1, #13, #7)	**Accession**	HQ672230	AACY023245853	NC_010793
	**Descrip.**	Uncultured bacterium clone F9P2610_S_P18	Marine metagenome	*Orientia tsutsugamushi*
	**Score**	2100	606	584
	**% ID**	93% (1347/1448)	77% (929/1208)	77% (925/1207)
**#9**	**Accession**	HQ672230	AACY020472379	NC_010793
	**Descrip.**	Uncultured bacterium clone F9P2610_S_P18	Marine metagenome	*Orientia tsutsugamushi*
	**Score**	1511	442	442
	**% ID**	93% (979/1055)	76% (733/962)	76% (733/962)
**#18**	**Accession**	HQ674331	AACY022560084	NC_008346.1
	**Descrip.**	Uncultured bacterium clone F9P262000_S_B04	Marine metagenome	*Syntrophomonas wolfei*
	**Score**	1941	1147	1026
	**% ID**	92% (1302/1421)	91% (785/862)	82% (1034/1255)
**#22**	**Accession**	HQ674331	AACY022560084	NC_008346
	**Descrip.**	Uncultured bacterium clone F9P262000_S_B04	Marine metagenome	*Syntrophomonas wolfei*
	**Score**	1949	1158	1026
	**% ID**	93% (1264/1364)	91% (786/861)	82% (1034/1255)

a: representative pf the cluster of unique sequences (after end-trimming) are noted in bold.
b: details of best BLASTn hit. Acc: subject database accession number; Des: description of the subject sequence; S: high-scoring segment pair (HSP) BLAST score; % ID: HSP percent identity to the query sequence.

Table S2a. Number of metagenome sequences and number of identified PHO4 sequences. Those under Total Pho4 were placed on the PHO4 tree (after retrieval using the HMM), but do not necessarily have statistical support for their respective placements. The under Euk/Virus could result from hosts or their respective viruses, while those in the viral column were unambiguously assigned to viruses. For both these categories only sequences retaining placement support (with probability ≥ 0.75) are included. Total non-redundant ORFs are based on 6-frame translation and minimum length of 40 and 60 amino acids for 454-FLX and Sanger, respectively. Breakdown of read numbers is given for all sequencing technologies in Table S4.

Sample Site	Total Reads	Total ORFs	Total Pho4	Euk/Virus	Virus
454-FLX					
H3 (5 m)	1,271,642	2,958,995	22	7	1
67-70 (10 m)	1,348,486	3,070,644	13	7	1
67-155 (5 m)	951,998	2,146,377	12	5	1
67-155 (86 m)	1,339,330	3,355,005	27	8	0
Sanger					
H3 (5 m)	33,731	185,977	5	1	0
67-70 (10 m)	215,973	388,870	12	1	0
67-155 (5 m)	-	-	-	-	-
67-155 (86 m)	166,361	1,010,666	4	0	0

Table S2b. Summary of PHO4 detected in the Global Ocean Sampling predicted protein datasets as deposited in CAMERA. All PHO4 were identified using the HMM and those in the columns Euk/Virus or Virus also had supported positions in the phylogenetic analysis (with probability ≥ 0.75). Sequences in Euk/Virus could result from hosts or their respective viruses, while those in the viral column were unambiguously assigned to viruses. Total reads does not reflected the number of predicted proteins and the number was not easily retrievable.

Ocean	Total Reads	Total PHO4	Euk/Virus	Virus	# Stations
**Atlantic**	5,249,354	1,176	125	34	21
**Pacific**	3,591,840	1,061	64	1	31
**Indian**	2,253,607	513	69	0	22

Table S3. Summary of PHO4 detected in the Monterey Bay line-67 sequence datasets for Sanger and 454-FLX sequencing technologies.

Sample Site	Size	Sanger Reads	Sanger ORFs	454 Reads	454 ORFs
	S	11,330	62,565	507,338	993,036
**H3 (5m)**	M	11,410	62,867	429,346	1,005,467
	L	10,991	60,545	334,958	960,433
	S	15,746	50,341	412,051	726,048
**67-70 (10m)**	M	100,238	642,775	499,086	1,446,345
	L	99,989	695,754	437,349	898,201
	S	-	-	387,007	703,403
**67-155 (5m)**	M	-	-	262,474	739,326
	L	-	-	302,517	703,650
	S	9,231	50,932	576,404	1,195,629
**67-155 (86m)**	M	103,490	663,630	284,359	789,542
	L	53,640	296,104	478,567	1,369,793

S=0.1μm to ≤0.8 μm; M= 0.8μm to ≤3μm; L= 3μm to ≤20μm

Monier et al Fig. S1

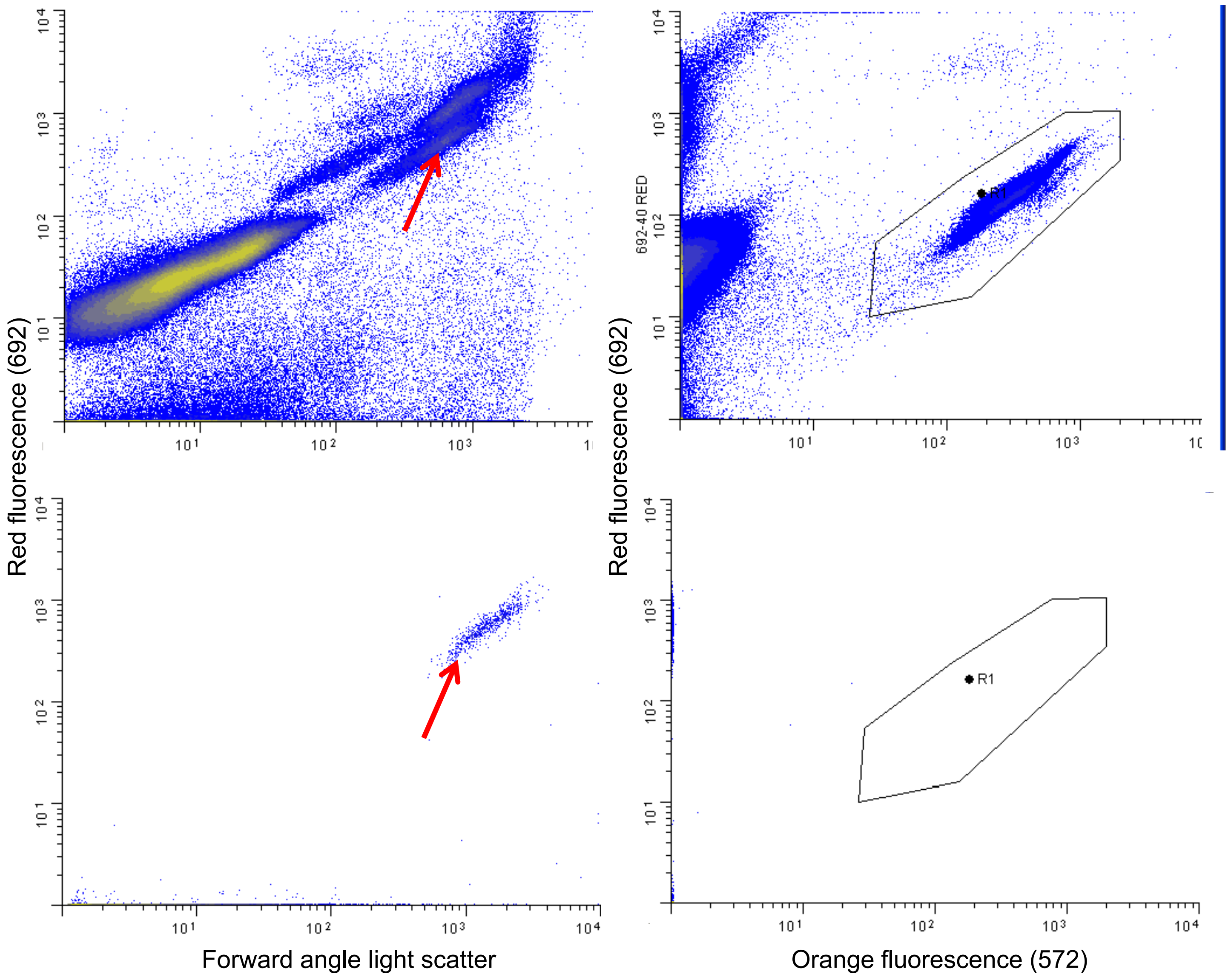

Fig. S1. Flow cytometry histograms of the pre-concentrated tropical Atlantic sample that was sorted. The arrow indicates the target populations. Top panels are the sample as it appeared at sea during sorting. The right hand panel shows *Synechococcus* cells (R1) which have been gated out from the left hand panel. The bottom panel shows the sorted population as thawed and rerun on land where it was resorted for purity. R1 again shows the position where *Synechococcus* would be expected. Note cytometer set up is never identical between runs (even if strived for) hence position of populations in top and bottom panels is slightly different.

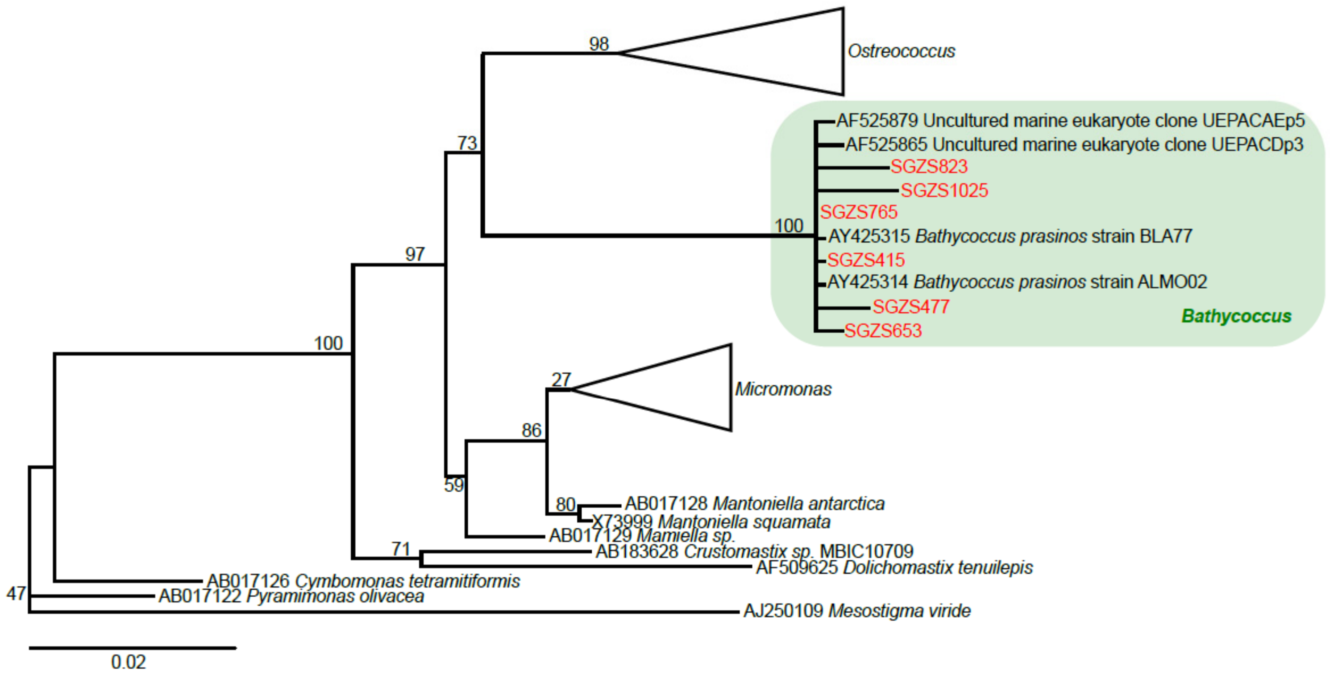

Fig. S2. Analysis of an 18S rDNA clone library from the sorted, MDA amplified population template. All of the 741 successfully sequenced 18S rDNA clones were phylogenetically placed with *Bathycoccus*. Prior to this maximum-likelihood analysis (PhyML) sequences were clustered at 99% identity (sequences were not manually curated) and a single representative of each of the 6 resulting clusters used in the alignment and tree. 830 homologous positions were analyzed after gap removal with 100 boot straps. After discarding gapped positions and ambiguous positions in the alignment , differences between *Bathyoccus* sequences were so few that it resulted in the observed polytomy in this reconstruction.

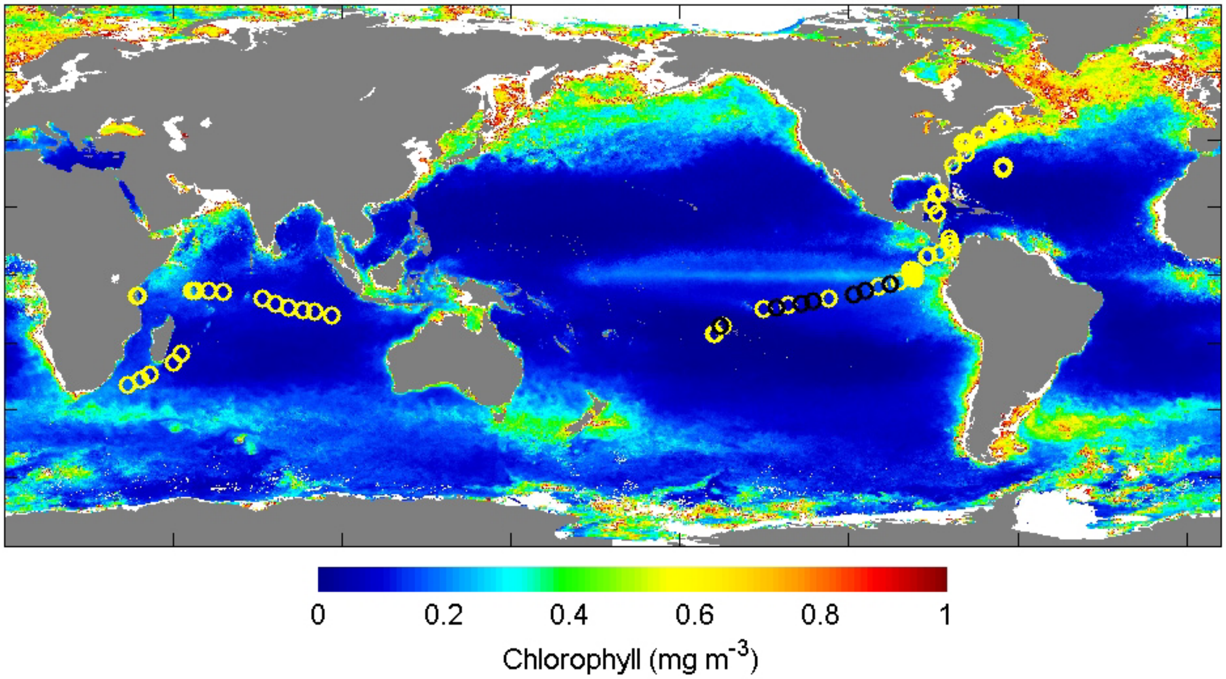

Fig. S3. Aqua MODIS chlorophyll concentration 1 February – 30 September 2005, the period spanning collection of sequenced GOS samples. Grey indicates land, white missing data, yellow circles indicate sites at which PHO4 was detected and black circles indicate those where no PHO4 were detected although the metagenome from the site was analyzed.
